# Editorial: Advances and Challenges of RNAi Based Technologies for Plants—Volume 2

**DOI:** 10.3389/fpls.2022.930851

**Published:** 2022-07-11

**Authors:** Bruno Mezzetti, Salvatore Arpaia, Elena Baraldi, Antje Dietz-Pfeilstetter, Guy Smagghe, Vera Ventura, Jeremy B. Sweet

**Affiliations:** ^1^Department of Agriculture, Food and Environmental Sciences, Marche Polytechnic University, Ancona, Italy; ^2^Agenzia Nazionale per le Nuove Tecnologie, l'Energia e lo Sviluppo Economico Sostenibile (ENEA) Research Centre Trisaia - Division Bioenergy, Biorefinery and Green Chemistry, Rotondella, Italy; ^3^DISTAL-Department of Agricultural and Food Science, Alma Mater Studiorum, University of Bologna, Bologna, Italy; ^4^Julius Kühn-Institut, Federal Research Centre for Cultivated Plants, Institute for Biosafety in Plant Biotechnology, Braunschweig, Germany; ^5^Laboratory of Agrozoology, Department of Plants and Crops, Ghent University, Ghent, Belgium; ^6^Department of Civil Engineering, Architecture, Land, Environment and of Mathematics, University of Brescia, Brescia, Italy; ^7^Sweet Environmental Consultant (SEC), Cambridge, United Kingdom

**Keywords:** agrifood, cross kingdom RNAi, sRNA, disease resistance, dsRNA, RNA products

The greatest challenge for farmers is to ensure sufficient and safe food in an economically, socially, and environmentally sustainable way. Plant pests and diseases are among the main problems affecting crop species, which are mainly controlled by the application of chemical pesticides (Schumann and D'Arcy, 2010). There is strong social and political pressure in Europe to decrease the use of agrochemicals, as also highlighted in the recently published European Green Deal, where a 50% decrease in the use of chemical pesticides is expected before 2030. However, recent estimations outline that the implementation of the EU policy can lead to a significant decline in EU agricultural production, together with a non-negligible increase in prices of agrifood products (Barreiro-Hurle et al., 2021): innovation is crucial to combine farm productivity with the goal of agrochemical reduction. One possible strategy is the employment of crop varieties resistant and/or tolerant to diseases through classical breeding techniques, but, besides the limited durability of this type of resistance, this solution is not always feasible due to the lack of resistance genes (McDonald, 2014; Willocquet et al., 2017).

The implementation of specific genetic improvement programs, including the application of new breeding techniques (NBTs), represents a faster and more precise strategy than conventional breeding for ensuring durable crop protection and yield improvement (Podevin et al., 2013; Limera et al., 2017; Sabbadini et al., 2021). As an additional tool, RNA interference (RNAi), an evolutionarily conserved regulatory process used by eukaryotes to regulate the expression of endogenous genes and also functioning in plants as a defense mechanism against pathogens, has been successfully exploited to confer disease protection and improve traits of agronomic interest (Limera et al., 2017; Rosa et al., 2022). Short-range movement of RNAi-mediating small non-coding regulatory RNAs (sRNAs) between cells occurs through plasmodesmata, whereas the long-distance movement occurs via the vascular systems (Tang et al., 2022). sRNAs can move between cells as naked molecules, bound to RNA binding proteins or enclosed in extracellular vesicles (EVs) (Tang et al., 2022).

The expression in plants of double strand RNA (dsRNA)-generating constructs permits the silencing of genes of invading pests and pathogens (host-induced gene silencing, HIGS) (Nowara et al., 2010). Two papers in this Research Topic are dealing with HIGS. Huang et al. (2022) used omics techniques to compare RNAi-based genetically modified maize lines resistant to a hemipteran insect with the parental line and found no specific unintended effects. The long-term efficacy and the stability of small RNA (sRNA) profiles of the RNAi-mediated virus resistant “Honeysweet” plum, as well as the absence of adverse health effects, is reported by Singh et al. (2021).

Most papers in this Research Topic are on exogenous application, usually by spraying, of sRNAs or dsRNAs on plant surfaces (spray-induced gene silencing, SIGS). The review *Lab-to-Field Transition of RNA Spray Applications – How far are we?* by Rank and Koch (2021) gives an overview on recent advances of exogenous RNA applications for the control of viruses, fungi and insects and for silencing of plant genes, and also points to challenges for agricultural uses of this approach.

A communication mechanism based on sRNA trafficking via EVs exchange between some fungal pathogens and their respective host plants, called bi-directional cross-kingdom RNAi which seems to modulate host immunity and pathogen virulence during infection processes, has recently been demonstrated (Wang et al., 2016; He et al., 2021). This discovery also enabled further developments of strategies for crop disease management based on SIGS.

Fungal/oomycete-associated diseases, including “gray mold” and “downy mildew” (caused by *B. cinerea* and *P. viticola*, respectively) are the most serious grapevine diseases worldwide, causing severe losses in crop yields and grape quality (Pearson and Goheen, 1988; Pons et al., 2018). They are generally controlled with a high use of pesticides, that in Europe is reaching the amount of 68,000 tons/year of fungicides to control grape diseases, equaling 65% of all fungicides used in agriculture (Eurostat Report, 2007).

Grape breeding programs aiming at the introgression of resistance traits from wild species to cultivated varieties have the limit to lose trait specificity of clones identified in important vine cultivars. Target sequences coding for Dicer and Dicer-like (*DCL)* proteins have been identified as crucial genes for the pathogenicity of *B. cinerea* and *P. viticola* and used in SIGS approaches to control these two severe pathogens (Wang et al., 2016; Capriotti et al., 2020; Haile et al., 2021). A SIGS approach against the oomycete *P. viticola* was shown to work effectively both as preventive and therapeutic treatment (Haile et al., 2021). A different approach for downy mildew control is presented by Marcianò et al. (2021) who silenced a grapevine susceptibility gene by exogenous dsRNA application, resulting in reduced disease severity after artificial *P. viticola* inoculation. In this study, control of downy mildew was achieved through the exogenously induced silencing of a plant-host gene, the *VviLBDIf7* gene encoding the putative ortholog of a transcription factor (TF) belonging to the LOB (lateral organ boundaries) family of TF, acting as repressor of jasmonate-mediated defense responses and known to be involved in plant organ development and stress response in many plant species. This evidence represents an alternative SIGS application, implying a deep knowledge of the genetic mechanism underneath the plant susceptibility and the need of new investigations to clarify any possible undesirable drawback on plant development and productivity. On the other hand, since susceptibility genes can be highly conserved among plant species and can work in response to different pathogens, once developed in a particular plant species, this approach can inspire alternative control strategies in various crop systems to provide broad protection.

Regarding the application of RNAi to control important pest insects, Willow and Veromann (2021) reviewed dietary RNAi studies in coleopterans. They discuss the impediments to our current understanding of RNAi sensitivity in this important insect order, and identify critical future directions for research in this area, with an emphasis on using plant biotechnology approaches. Joga et al. (2021) report that RNAi is an emerging approach that can be used for forest protection. Forests are of immense importance due to their socio-economic and ecosystem services, and Coleopteran Forest pests such as Emerald ash borer (*Agrilus planipennis*), Asian longhorn beetles (*Anoplophora glabripennis*) and bark beetles have taken advantage of ongoing climate change, causing severe damage to the forests worldwide. Current management strategies have been unable to keep pace with these forest insect population infestations. Joga et al. (2021) focus in their review on recent innovations in RNAi delivery that can be deployed against forest pests, for instance cationic liposome-assisted (lipids), nanoparticle-enabled (polymers or peptides), symbiont-mediated (fungi, bacteria, and viruses), and plant-mediated deliveries (trunk injection, root absorption). They consider barriers to further developing RNAi for forest pest management and suggest future directions of research in order to develop the use of RNAi against wood-boring coleopterans.

One of the most alluring aspects of RNAi technology is its predicted minimal impact on the environment, due to high target selectivity and the short persistence of the active molecules in the environment. However, in order to exclude possible off-target effects and effects on non-target organisms and due to the limited knowledge of most eukaryotic genomes, the combination of bioinformatics and ecologically sound bioassays using selected focal species are deemed necessary to support biosafety claims of RNAi applications (Christiaens et al., 2022). In addition, it should be noted here that the exploitation of RNAi to improve crop health is a fast-growing market and while GM RNAi plants are being assessed using the existing regulatory framework, appropriate safety evaluations, and authorization procedures for SIGS-based products are less clear so far (De Schutter et al., 2022). Externally applied RNA products must be regulated according to pesticide regulations. Regulatory frameworks for plant protection products and considerations for the authorization of dsRNA-based pesticides for plant protection in the United States and in the European Union are outlined by Dietz-Pfeilstetter et al. (2021).

## Author Contributions

All authors listed have made a substantial, direct, and intellectual contribution to the work and approved it for publication.

## Funding

BM received funding from the MIUR-PRIN2017 national program via Grant No. 20173LBZM2-Micromolecule.

## Conflict of Interest

The authors declare that the research was conducted in the absence of any commercial or financial relationships that could be construed as a potential conflict of interest.

## Publisher's Note

All claims expressed in this article are solely those of the authors and do not necessarily represent those of their affiliated organizations, or those of the publisher, the editors and the reviewers. Any product that may be evaluated in this article, or claim that may be made by its manufacturer, is not guaranteed or endorsed by the publisher.
